# The maturation of auditory responses in infants and young children: a cross-sectional study from 6 to 59 months

**DOI:** 10.3389/fnana.2015.00131

**Published:** 2015-10-16

**Authors:** J. Christopher Edgar, Rebecca Murray, Emily S. Kuschner, Kevin Pratt, Douglas N. Paulson, John Dell, Rachel Golembski, Peter Lam, Luke Bloy, William Gaetz, Timothy P. L. Roberts

**Affiliations:** ^1^Lurie Family Foundations MEG Imaging Center, Department of Radiology, Children’s Hospital of PhiladelphiaPhiladelphia, PA, USA; ^2^Tristan Technologies, Inc.San Diego, CA, USA

**Keywords:** infant, young child, magnetoencephalography, auditory

## Abstract

**Background**: An understanding of the maturation of auditory cortex responses in typically developing infants and toddlers is needed to later identify auditory processing abnormalities in infants at risk for neurodevelopmental disorders. The availability of infant and young child magnetoencephalography (MEG) systems may now provide near optimal assessment of left and right hemisphere auditory neuromagnetic responses in young populations. To assess the performance of a novel whole-head infant MEG system, a cross-sectional study examined the maturation of left and right auditory cortex responses in children 6- to 59-months of age.

**Methods**: Blocks of 1000 Hz (1st and 3rd blocks) and 500 Hz tones (2nd block) were presented while MEG data were recorded using an infant/young child biomagnetometer (Artemis 123). Data were obtained from 29 children (11 males; 6- to 59-months). Latency measures were obtained for the first positive-to-negative evoked response waveform complex in each hemisphere. Latency and age associations as well as frequency and hemisphere latency differences were examined. For the 1000 Hz tone, measures of reliability were computed.

**Results**: For the first response—a response with a “P2m” topography—latencies decreased as a function of age. For the second response—a response with a “N2m” topography—no N2m latency and age relationships were observed. A main effect of tone frequency showed earlier P2m responses for 1st 1000 Hz (150 ms) and 2nd 1000 Hz (148 ms) vs. 500 Hz tones (162 ms). A significant main effect of hemisphere showed earlier N2m responses for 2nd 1000 Hz (226 ms) vs. 1st 1000 Hz (241 ms) vs. 500 Hz tones (265 ms). P2m and N2m interclass correlation coefficient latency findings were as follows: left P2m (0.72, *p* < 0.001), right P2m (0.84, *p* < 0.001), left N2m (0.77, *p* < 0.001), and right N2m (0.77,*p* < 0.01).

**Conclusions**: Findings of strong age and latency associations, sensitivity to tone frequency, and good test-retest reliability support the viability of longitudinal infant MEG studies that include younger as well as older participants as well as studies examining auditory processing abnormalities in infants at risk for neurodevelopmental disorders.

## Introduction

Electroencephalography (EEG) studies have identified characteristic patterns of auditory long-latency responses in infants, with full-term neonates demonstrating a midline negativity that soon becomes a positive component (Kurtzberg et al., 1984). This midline positivity is sometimes referred to as the infantile P2 (Graziani et al., 1974; Barnet, 1975; Ohlrich et al., 1978; Rotteveel et al., 1987; Shucard et al., 1987). During development, a response of the opposite polarity is initially observed as a discontinuity in the P2 (Novak et al., 1989). Although there is nomenclature variability in the literature, with some groups referring to this opposite polarity response at about 200 ms as N1 (Barnet, 1975; Ohlrich et al., 1978; Novak et al., 1989) and others as N2 (Rotteveel et al., 1987; Shucard et al., 1987), the auditory event-related potential (ERP) is dominated by this positive-to-negative waveform morphology in infants and young children (Ceponiene et al., 2002; Kushnerenko et al., 2002).

Across infant and young child development there is rapid change in the latency of auditory responses. As an example, Barnet (1975) examined auditory responses (EEG electrode Cz) during sleep in 130 infants and children between the ages of 10 days and 37 months. Cross-sectional findings showed that over a 3-year period the latency decrease for P2 was approximately 75 ms and for N2 215 ms. A few longitudinal studies have studied also the maturation of auditory responses in infants and young children (Novak et al., 1989; Kushnerenko et al., 2002; Choudhury and Benasich, 2011). For example, Ohlrich et al. (1978) examined changes in auditory responses (EEG Cz) in 16 typically developing children tested repeatedly between the ages of 2 weeks and 3 years. Consistent with the cross-sectional studies, significant changes in P2 and N2 latency were observed.

Although the majority of electrophysiological studies examining cortical auditory responses in infants and young children have used EEG, a few more recent studies have used adult magnetoencephalography (MEG) systems (Bosseler et al., 2013; Kuhl et al., 2014) and more recently, infant and young child whole-head MEG systems (Yoshimura et al., 2012; Roberts et al., 2014). For examining cortical auditory activity, MEG is sometimes preferred over EEG as the superior temporal gyrus (STG) auditory generators are favorably positioned to provide distinct measures of left and right STG activity given MEG’s selective sensitivity to superficial tangentially-oriented neural currents and thus spatially-separated left and right auditory neuromagnetic fields (Edgar et al., 2003), even in infants and children (Paetau et al., 1995; Huotilainen et al., 2008). MEG is also sometimes preferred in studies examining neural brain activity in infants and young children as MEG is much less sensitive than EEG to distortions of the volume current caused by the incompletely developed fontanels and sutures in young populations and thus to inaccurate estimates of skull conductivity (Lew et al., 2013). Finally, MEG is also much less sensitive to conductivity differences between the brain to cerebral spinal fluid to skull to scalp, and thus for source localization MEG is sometimes preferred over EEG (Hämäläinen et al., 1993). Despite the difficulties examining auditory responses in infants using EEG, dense-array EEG and advanced source localization procedures (co-registering to age-appropriate MRI templates and using age-specific EEG conductivity measures) have been used to study the neural mechanisms associated with the acquisition of language in infants (Ortiz-Mantilla et al., 2012, 2013; Musacchia et al., 2013).

A limitation of conventional MEG is the fixed adult-sized sensor helmet, resulting in a substantial distance between brain neural generators and MEG detection coils in infants and young children, with this increased distance resulting in less-than-optimal MEG recordings due to a loss of brain signal as a function of (the square of the) distance. To address this limitation, whole-head infant MEG system optimized for children have been developed (see reviews in Gaetz et al., 2015; Kikuchi et al., 2015). A few whole-head infant MEG studies have examined auditory neural responses in infants and young children (Yoshimura et al., 2012, 2013). For example, in a longitudinal infant MEG study, Yoshimura et al. (2014) measured auditory P1 m responses in twenty TD children at 36–75 months and then again 11–25 months later. A primary finding was that left hemisphere P51 m amplitude change was associated with positive change on a language conceptual inference task.

The present study used a recently developed whole-head MEG system—Artemis 123—with a helmet designed for the median 3-year-old head circumference and thus ideal for studying infants and children given a decreased distance between neural brain activity and MEG detectors (Roberts et al., 2014). The present study reports on the sensitivity and reliability of the left and right auditory measures obtained using this novel MEG system, examining children aged 6- to 59-months. Based on prior findings, quality MEG auditory recordings would be demonstrated via the following findings: (1) the latency of the prominent positive-to-negative auditory response would decrease with age (left and right hemisphere auditory measures); (2) left and right auditory responses would be earlier to higher frequency (1000 Hz) vs. lower frequency tones (500 Hz), reflecting tonochronic principles (Roberts and Poeppel, 1996; Roberts et al., 1998, 2000); and (3) the latency of the response to the 1st and 3rd 1000 Hz tone blocks would be similar, indicating that the MEG obtained auditory measures are reliable. Positive findings would indicate that Artemis 123 provides quality primary/secondary auditory cortex recordings in infants and young children.

## Materials and Methods

### Participants

Participants were selected according to the following criteria: (1) between 6 and 60 months old; (2) speak/hear English as their first language; (3) no seizure disorder in the child or any immediate family members; (4) no premature birth; (5) no non-removable metal in the body; (6) no known genetic conditions or neurological disorders; (7) no known hearing loss; and (8) no language or developmental delay concerns. Participants were included or excluded based on parental report via a phone screen. The study was approved by the Children’s Hospital of Philadelphia IRB and all participants’ families gave written consent.

Thirty-six children meeting study inclusion and exclusion criteria were enrolled. Of the enrolled children, MEG data were not obtained from four children because they were not able to place (or keep) their head in the MEG helmet: one 9-month-old male, one 10-month-old male, one 16-month-old female, and one 48-month-old male. MEG data were obtained but unusable from three children due to muscle artifact or technical problems: one 7-month-old female, one 10-month-old female, and one 12-month-old male. Of the remaining 29 participants, a full dataset (1000 Hz 1st, 500 Hz 1st, 1000 Hz 2nd) was obtained from 26 participants. A partial dataset (1000 Hz 1st, 500 Hz 1st,) was obtained from the remaining three participants. In the final group of 29 participants, ages ranged from 6- to 59-months, with a mean age of 27.2 months (SD = 17.0). In the 6–11 month age group there were four males and four females, in the 12 month to 23 month age group no males and five females, in the 24–35 month age group three males and four females, in the 36–47 month age group one male and two females, and in the 48–59 month age group four males and two females.

### MEG Data Acquisition

Whole-head MEG data were recorded using Artemis 123 (Tristan Technologies, San Diego, CA, USA) with a sampling rate of 5000 Hz and a 0.1–330 Hz bandpass. Stimuli consisted of 500 and 1000 Hz tones of 300 ms duration and 10 ms rise time. Stimuli were presented via a free field speaker at 80 dB SPL (Panphonics Sound Shower). Exams were blocked, presenting 1000 Hz tones (1000 Hz 1st), then 500 Hz tones (500 Hz 1st), and then a repeated block of 1000 Hz tones (1000 Hz 2nd). All tones were presented with a 2100 ms (±500 ms) inter-trial interval. As sleep can have an effect on the amplitude and latency of auditory responses (Weitzman et al., 1965; Barnet, 1975; Picton et al., 1981), recording were obtained only during an awake state. A variety of strategies were used to keep infants still and awake, including projecting a preferred video on the ceiling, toys to maintain their attention and focus (e.g., finger puppets, books, visually interesting sensory toys), and using a pacifier.

MEG data were analyzed using BESA 6.0 (BESA Gmbh). MEG data were downsampled to 300 Hz. Epochs with large amplitude or gradient artifacts were manually rejected. The number of artifact-free trials per condition was: 1000 Hz 1st mean = 117 (range 53–206); 500 Hz mean = 119 (range 30–194), and 1000 Hz 2nd mean = 119 (range 82–158). Epochs 200 ms pre- to 400 ms post-stimulus were defined from the continuous recording, artifact-free epochs averaged according to stimulus type, and a 2–55 Hz bandpass filter applied. In almost all participants, an auditory response with a positive topography was observed at approximately 150 ms, followed by a response with an opposite field pattern. Given similar findings from previous studies (see “Introduction” Section), the present study focused on this positive-to-negative evoked complex. Kushnerenko et al. (2002) noted that during early maturation an initial broad positivity in infants is divided into two positive peaks by the emergence of a growing negativity (N250), sometimes referred to as the P150 and the P350. In the present study, the first positivity preceding the N2 was measured. As in Barnet (1975), in the present study, these “positive” and “negative” responses are referred to as P2 and N2, although adding an “m” to obtain P2m and N2m, as the recorded activity is magnetic rather than electric (for a similar approach, see also the language acquisition studies conducted by Rivera-Gaxiola et al. (2005, 2007, 2012) examining the P150–250 and N250–550 in infants).

**Figure 1:**
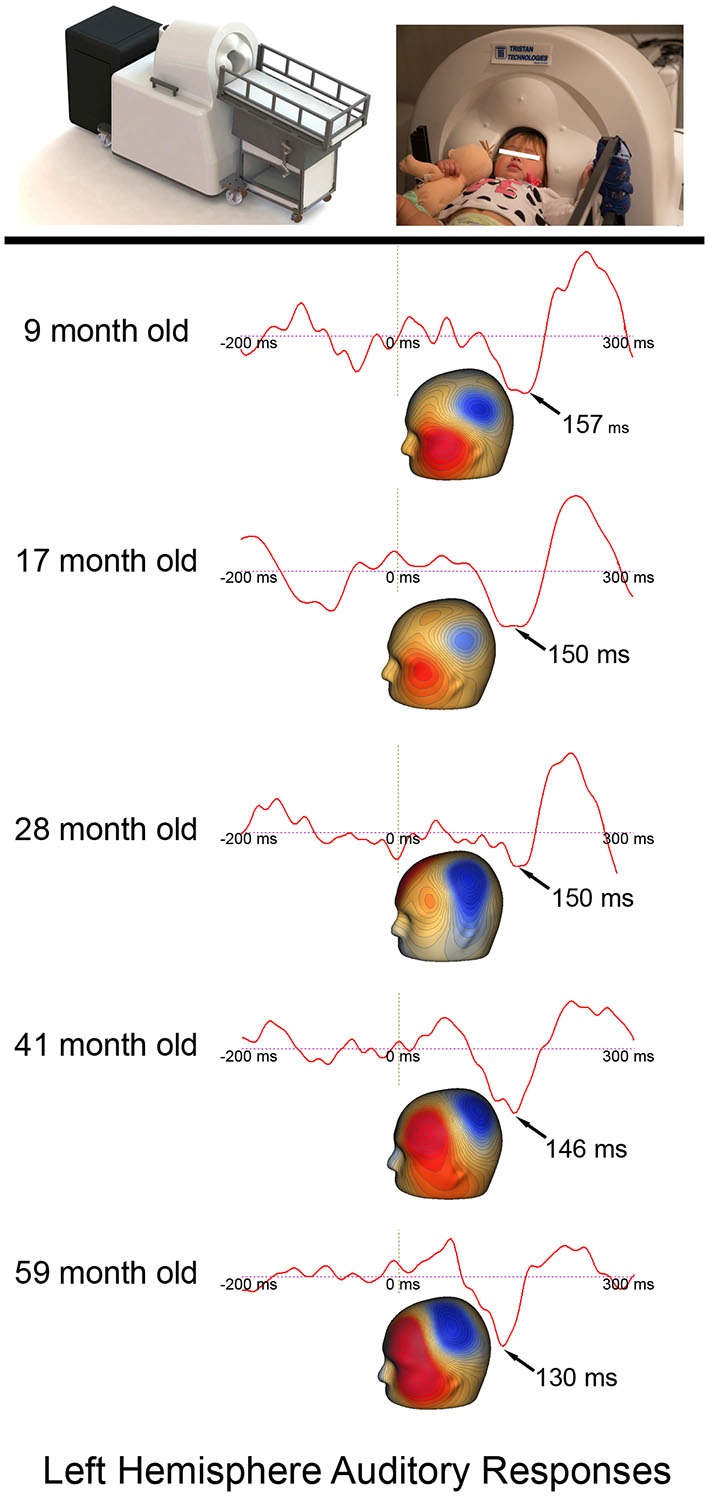
**The Artemis 123 system and a child placed in the helmet, along with left hemisphere auditory responses from five participants ranging in age from 6- to 59-months-old.** The expected earlier P2m (magnetic field topography shown) auditory latencies in older (~130 ms) vs. younger children (~157 ms) is observed. Permission was granted by the family to publish the participant’s photograph in the top panel.

Given that continuous head position indicator (HPI) information was available only for the last few participants, the following procedure was used to obtain left and right P2m and N2m latency measures. First, after excluding all MEG sensors opposite the hemisphere of interest, P2m and N2m latencies were obtained from the 1st component derived from a principal components analysis (PCA) applied to the sensor data. In all participants, field topography was examined to ensure that the P2m response and the N2m response had the characteristic magnetic field pattern. N2m was first identified, with P2m operationally defined as the first preceding response showing a reversed field pattern. Figure 1 shows example timecourse waveforms associated with the 1st PCA component for a subject in each age group.

## Results

### P2m and N2m Latency and Age Associations

**Figure 2:**
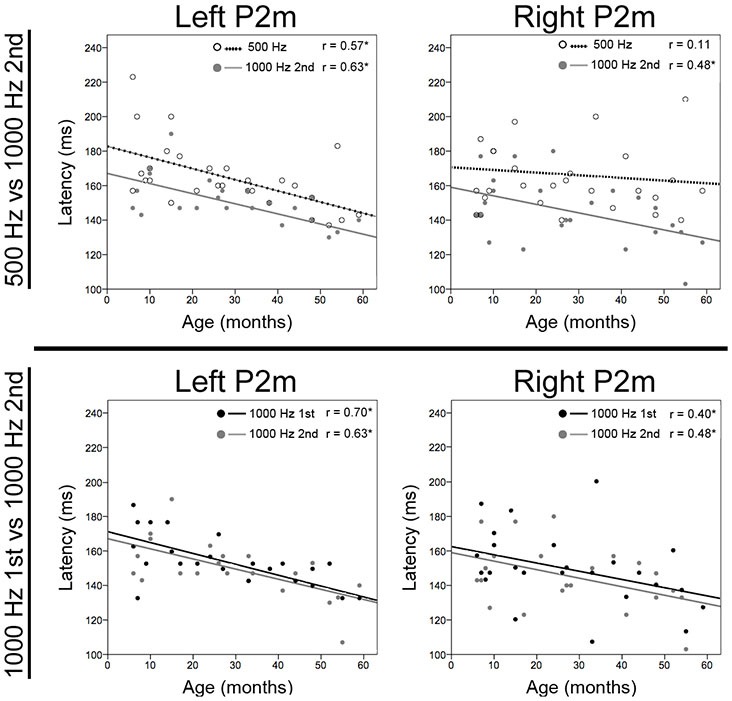
**Scatterplots showing associations for each tone between age and left and right P2m latency.** The top row shows associations for 500 Hz (open circle and dotted line) and 1000 Hz 2nd tones (gray circle and line), and the bottom row shows associations for 1000 Hz 1st (black circle and line) and 1000 Hz 2nd tones. The *x* axis shows age and the *y* axis latency. Significant correlations are marked with an “*”.

Correlations examined associations between P2m and N2m latency with age for each tone and hemisphere. As shown in Table 1, age and P2m latency associations were observed for all tones and in both hemispheres except right 500 Hz, with a slope of −0.6 ms/month observed for most conditions. Age and N2m latency associations were not observed for any condition. The Figure 2 top row scatterplots compare age and latency associations for 500 and 1000 Hz 2nd tones. The Figure 2 bottom row scatterplots compare age and latency associations for the 1000 Hz 1st and 1000 Hz 2nd tones.

**Table 1:** **P2m and N2m latency associations with age**.

	P2m			N2m		
	1st 1000 Hz *r*-value and slope	500 Hz *r*-value and slope	2nd 1000 Hz *r*-value and slope	1st 1000 Hz *r*-value and slope	500 Hz *r*-value and slope	2nd 1000 Hz *r*-value and slope
Left	0.70** (−0.6 ms/month)	0.57** (−0.6 ms/month)	0.63** (−0.6 ms/month)	0.11 (0.2 ms/month)	0.11 (−0.2 ms/month)	0.27 (−0.2 ms/month)
Right	0.40* (−0.5 ms/month)	0.11 (−0.2 ms/month)	0.48* (−0.5 ms/month)	0.17 (0.3 ms/month)	0.30 (0.6 ms/month)	0.30 (−0.6 ms/month)

*p < 0.05, **p < 0.01.

### P2m and N2m Latency

Repeated measures ANOVA examined tone (1000 1st, 500 1st, and 1000 2nd) and hemisphere differences in P2m and N2m latency. Table 2 shows P2m and N2m mean and standard deviation latency values for each condition. For P2m, a main effect of tone, *F*_(2,18)_ = 7.18, *p* < 0.01, showed earlier P2m responses for 1st 1000 Hz (150 ms) and 2nd 1000 Hz (148 ms) vs. 500 Hz tones (162 ms). For N2m, a main effect of tone, *F*_(2,18)_ = 18.35, *p* < 0.001, showed earlier N2m responses for 2nd 1000 Hz (226 ms) vs. 1st 1000 Hz (241 ms) vs. 500 Hz tones (265 ms). No hemisphere or tone by hemisphere effects were observed for P2m or N2m (*p*’s > 0.19).

**Table 2:** **P2m and N2m latency**.

	P2m			N2m		
	1st 1000 Hz Latency (ms) and SD	500 Hz Latency (ms) and SD	2nd 1000 Hz Latency (ms) and SD	1st 1000 Hz Latency (ms) and SD	500 Hz Latency (ms) and SD	2nd 1000 Hz Latency (ms) and SD
Left	154 (15)	163 (20)	149 (16)	230 (25)	263 (28)	222 (22)
Right	149 (21)	163 (19)	146 (22)	242 (40)	268 (38)	229 (41)

### P2m and N2m Reliability

To examine the similarity of the auditory measures, correlation analyses examined associations between P2m and N2m latencies to the 1000 Hz 1st and 1000 Hz 2nd tones. Left P2m latencies (*r* = 0.57, *p* < 0.01) and right P2m latencies were significantly associated (*r* = 0.76, *p* < 0.001). Left N2m latencies (*r* = 0.62, *p* < 0.01) and right N2m latencies were also significantly associated (*r* = 0.71, *p* < 0.001). Intraclass correlation coefficient (ICC) findings were as follows: left P2m (0.72, *p* < 0.001), right P2m (0.84, *p* < 0.001), left N2m (0.77, *p* < 0.001), and right N2m (0.77, *p* < 0.01).

## Discussion

Strong cross-sectional P2m age and latency associations, robust findings of sensitivity to 500 Hz vs. 1000 Hz tone frequency, and good reliability measures (0.70–0.84), all support the viability of infant and young child Artemis 123 MEG auditory studies. Below, present findings are discussed within the context of previous findings.

Although direct comparisons to previous studies are difficult given that many earlier studies examined infants and children during sleep (Ohlrich et al., 1978), examined auditory responses at a single midline EEG electrode (Barnet, 1975; Ohlrich et al., 1978), or used an inter-stimulus interval shorter than that used in the present study Sharma et al. (2002), present findings were generally consistent with previous studies. For example, Ohlrich et al. (1978) reported a P2 latency of 153 ms in 3-year-old children, and Barnet (1975) a P2 latency between 150 and 170 in children between the ages of 1.5–3 years. Examining 4-year-old children, Ceponiene et al. (2002) observed a P1 latency of 114 ms and a N2 latency of 295 ms (midline EEG electrodes). Examining the auditory Cz response in 3- and 4-year-olds using a 2000 ms ISI, Gilley et al. (2005) reported a P2 latency of 145 ms. Using high-density EEG and assessing source localized left and right STG activity, Ortiz-Mantilla et al. (2013) observed in 6-month-olds a grand average left P1 latency of 176 ms and right P1 latency of 208 ms in response to consonant-vowel syllable stimuli and presenting stimuli at an approximately 700 ms ISI. Latencies in the present study, however, were somewhat longer than the P150 latencies of 139 ms in 9-month-olds and 142 ms in 12-month-olds reported in Kushnerenko et al. (2002), and the P1 latencies of 150 ms in 12-month-olds and 130 ms in 36-month-olds reported in Choudhury and Benasich (2011), with study differences perhaps due to the use of a shorter ISI in these two studies vs. the present study.

In the present study, a latency decrease of 0.6 ms/month was observed for P2m (i.e., 7.2 ms/year), with 1000 Hz P2m latencies estimated to be approximately 170 ms at 6 months and 130 ms at 5 years. These rate-of-change estimates are again generally consistent with prior studies (Ohlrich et al., 1978; Novak et al., 1989). For example, in a longitudinal study following children from 6 months to 4 years, Choudhury and Benasich (2011) reported age-related decreases for the positive-going peaks of 20–80 ms over 4 years, and a decrease for the negative peaks of 9–50 ms over 4 years.

Infant and young child auditory responses differ from adult responses, and there is evidence that the P2m response examined in the present study eventually “becomes” the adult P50/M50 (P1) response (Kushnerenko et al., 2002; Ceponiene et al., 2003, 2008). The 1.9 ms/year rate-of change for P1 (EEG Fz) reported in Sharma et al. (1997) is slower than the rate-of-change observed in the present sample, suggesting more rapid changes to P2m earlier in development, and then with slower although continued changes through childhood, and with adult-like STG P50/M50 latencies not observed until late adolescence.

The first negative component identified in infants is often labeled the N1, N250, or N2. The literature suggests that the N2m component does not develop into the adult M100 response. For example, in a longitudinal study (birth to 12 months), using harmonic tones, the infant N1 was identified at birth and showed consolidation by 6 months of age (Kushnerenko et al., 2002). The authors found that the infant N1 component did not change in latency from birth until 12 months of age, and they thus suggested that the infant N1 may be a correlate of the childhood N250. Similar findings have been reported in other studies (Pang and Taylor, 2000; Ponton et al., 2000). In the present study, the finding that N2m latency did not change as a function of age in children 6 months and older is consistent with previous studies (Onishi and Davis, 1969; Tanguay et al., 1973; Kushnerenko et al., 2002; Choudhury and Benasich, 2011), including observations from other studies showing that the adult N100/M100 is not robustly observed until early adolescence (Ponton et al., 2000, 2002; Edgar et al., 2014).

Previous studies have reported hemisphere differences in the maturation of left and right STG auditory cortex (Gomes et al., 2001). Although in older children and in adults earlier right than left M50 latencies are observed, in the present study, no P2m or N2m hemisphere latency differences were observed. Paetau et al. (1995) observed that in children 3–15 years auditory latencies tended to be earlier over the right than left hemisphere only in the older children. Examining EEG source localized left and right STG activity in 4-month-old infants, Musacchia et al. (2013) observed earlier right than left “P1” responses. The (Musacchia et al., 2013) inter-trial interval was much shorter than in the present study, perhaps suggesting that a right hemisphere auditory encoding advantage may be observed only under more demanding encoding conditions. Longitudinal studies following children from infancy through early adolescence are needed to better understand the development of infant and young child P2m responses and transition to adult M50 responses.

Changes to the auditory response (morphology and latency) as a function of age are hypothesized to be due to maturation of gray and white matter structure, such as age-related changes in synaptic efficiency (Goodin et al., 1978; Eggermont, 1988), cortical layer maturational changes (Steinschneider et al., 1994; Eggermont and Ponton, 2003). Maturational changes to the morphology of primary auditory cortex pyramidal cells are also observed (Elston et al., 2010; Elston and Fujita, 2014), with such changes of interest as MEG recordings primarily reflect pyramidal cell activity (Lewine and Orrison, 1995; Spruston, 2008). Multimodal human *in vivo* imaging studies are beginning to demonstrate how brain structure is related to brain function. For example, multimodal MEG and diffusion MR children and adolescent studies have shown that the latency of auditory responses decreases as a function of maturation of local white matter myelination (Roberts et al., 2009, 2013). In adults, studies have noted associations between gray matter cortical thickness and the strength of auditory responses (Edgar et al., 2012). Studies examining auditory cortex function and structure associations in infants and young children are now needed.

A few limitations of the present study are of note. First, given that HPI coil information was not available from most of the participants, it was not possible to determine the exact location of the infant or child’s head in the helmet and thus not possible to accurately estimate the strength of auditory responses. As such, although age-related auditory component amplitude changes are of interest in infants and young children (Ponton et al., 2002; Gilley et al., 2005; Yoshimura et al., 2014), examination of amplitude and age associations was not possible in the present study. Second, auditory responses in infants and children likely depend on the type of stimuli such as speech or non-speech stimuli (Friedman et al., 1984; Kurtzberg et al., 1984; Pang and Taylor, 2000), with present findings thus limited in their generality. Finally, a limitation of the present study is that acquisitions, and thus analyses, were cross-sectional rather than longitudinal.

## Conclusion

Strong cross-sectional P2m age and latency associations, robust findings of sensitivity to tone frequency, and good reliability measures all support the viability of infant and young child Artemis 123 MEG auditory studies.

## Funding

This research was supported by grants from the Nancy Lurie Marks Family Foundation, a IDDRC grant to CHOP (U54 HD08694), and the Pennsylvania Department of Health. The Pennsylvania Department of Health specifically disclaims responsibility for any analyses, interpretations or conclusions.

## Conflict of Interest Statement

The Associate Editor Dr. Huang declares that, despite being affiliated to the same university as the authors, the review process was handled objectively. The authors declare that the research was conducted in the absence of any commercial or financial relationships that could be construed as a potential conflict of interest.
